# An ultrasonographic tumor location-based model for predicting central lymph node metastasis in unifocal papillary thyroid microcarcinoma

**DOI:** 10.3389/fendo.2026.1785660

**Published:** 2026-05-14

**Authors:** Nan Pang, Ranglamu Cai, Wenhui Zhang, Ruoyao Zhang, Qian Sun, Yuyuan Huang, Jie Zhang

**Affiliations:** 1 Department of General Surgery, Tianjin Medical University General Hospital, Tianjin, China; 2 Tianjin Key Laboratory of Precise Vascular Reconstruction and Organ Function Repair, Tianjin, China; 3 Tianjin General Surgery Institute, Tianjin, China; 4 Baodi District Hospital of Traditional Chinese Medicine, Tianjin, China; 5 Ultrasound Department Affiliated Hospital of North China University of Science and Technology, Hebei Tangshan, China

**Keywords:** central lymph node metastasis, nomogram, papillary thyroid microcarcinoma, spatial location, ultrasound

## Abstract

**Objective:**

Accurate preoperative assessment of central lymph node metastasis (CLNM) in patients with unifocal papillary thyroid microcarcinoma (PTMC) remains challenging. This study aimed to develop and validate a nomogram integrating tumor spatial location with clinical and ultrasonographic features for predicting central lymph node metastasis (CLNM).

**Methods:**

In this retrospective study, 432 patients with surgically confirmed unifocal PTMC were enrolled. Independent predictors were identified through univariate and multivariate regression, and a nomogram was constructed and internally validated.

**Results:**

Age ≤38 years, tumor diameter >0.95 cm, microcalcifications, suspicious central/lateral lymph nodes, tumor in the isthmus, and tumor in the middle/inferior pole adjacent to the posterior capsule were independent risk factors for CLNM, while Hashimoto’s thyroiditis was protective. The nomogram showed good discrimination, with AUCs of 0.772 (training) and 0.728 (validation). Calibration and decision curve analysis confirmed clinical utility.

**Conclusion:**

The spatial location-based nomogram provides an effective preoperative tool for quantifying CLNM risk, assisting in individualized surgical planning and reducing overtreatment in unifocal PTMC.

## Introduction

The global incidence of papillary thyroid carcinoma (PTC) has risen significantly, largely attributable to the widespread use of high-resolution ultrasonography and other imaging techniques (1). This increase is primarily driven by a sharp rise in the incidence of PTMC (2, 3). Although most PTMCs progress slowly and often exhibit indolent behavior, the rate of CLNM ranges from 30.3% to 61.2%, significantly impacting patient prognosis (4–7). Studies show that the recurrence risk in PTMC patients with CLNM is three times higher than in those without metastasis (5). Consequently, determining the precise indication and extent of central lymph node dissection (CLND) is a critical clinical challenge.

However, CLND, as an invasive procedure, can cause serious complications such as recurrent laryngeal nerve injury and permanent hypoparathyroidism. While therapeutic CLND is indicated for patients with clinically evident metastasis, the role of routine prophylactic CLND remains controversial because it does not significantly reduce the risk of local recurrence in patients without definitive evidence of metastasis (8, 9). Therefore, accurate preoperative assessment of actual pathological CLNM status is particularly important for optimizing patient management, reducing surgery-related complications, and avoiding overtreatment.

In recent years, active surveillance (AS) and ablation therapy have gradually gained acceptance as management strategies for PTMC. The American Thyroid Association formally included ablation therapy in the PTMC management guidelines released in 2025(Recommendation 11) (10). AS can effectively alleviate healthcare burdens and prevent overtreatment. In Japan, the pioneer in proposing and implementing AS, the cost of the surgery group was approximately 4.1 times that of the AS group, allowing patients to maintain a good quality of life at low cost (2, 11). Ablation therapy can significantly alleviate patient anxiety related to “living with cancer,” surgical scars, and potential complications, making it more popular among younger patients (12). It is important to note that AS and ablation therapy are strictly indicated only for true T1aN0M0 cases. Because CLNM is an absolute contraindication for these conservative approaches (2, 10, 13, 14), accurately predicting the true pathological lymph node status in patients with unifocal PTMC before surgery is crucial to avoid inappropriate application of AS or ablation.

Ultrasonography is the primary method for preoperative assessment of CLNM. However, interference from thyroid gland coverage and anatomical structures like the trachea results in low sensitivity (<50%) of conventional ultrasound for CLNM. The accuracy of ultrasound combined with computed tomography (CT) is only about 70% (15). Moreover, given the extremely low rate of distant metastasis in low-risk PTMC, routine chest CT may do more harm than good (16–18). Previous studies have suggested associations between CLNM in PTMC and factors such as gender, age, tumor size, extrathyroidal extension, multifocality, tumor location, and ultrasonographic feature (19–22), but the results have been inconsistent. Therefore, based on PTMC tumor location, we combined clinical and ultrasonographic features to perform factor analysis and construct a risk prediction model, aiming to provide a reference for the individualized diagnosis and treatment of patients with unifocal PTMC. To ensure data homogeneity and quality control, this study was conducted within a single high-volume tertiary center following standardized protocols.

## Materials and methods

### Patients

**Figure 1:**
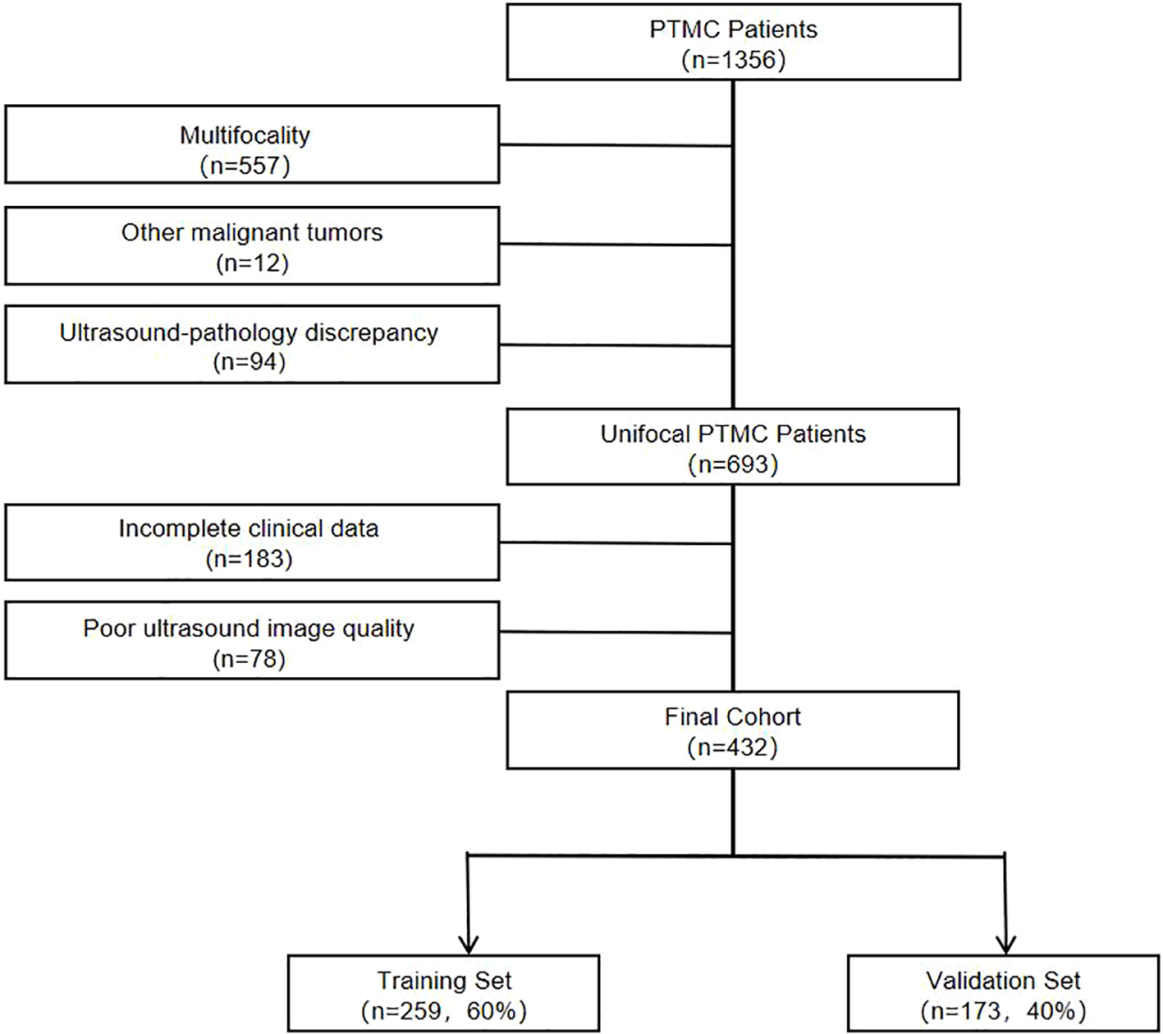
Patient selection flowchart. after applying exclusion criteria, 432 patients with unifocal PTMC were enrolled. They were randomly assigned to a training set and a validation set in a 6:4 ratio.

A retrospective analysis was conducted on 432 patients with pathologically confirmed unifocal PTMC who underwent thyroidectomy with central lymph node dissection at Tianjin Medical University General Hospital between January 2019 and July 2024 (**Figure 1**).

The inclusion criteria were (1): Age ≥18 years (2); Pathological confirmation of unifocal PTMC (3); Undergone neck lymph node dissection (4); No prior history of thyroid surgery or concurrent other malignancies story (5); Complete preoperative ultrasound examination data with clear images (6); Complete clinical, sonographic, and pathological records. The exclusion criteria were: (1) Preoperative radiotherapy, chemotherapy, radiofrequency ablation, or other neoadjuvant therapies; (2) No preoperative thyroid ultrasound examination; (3) Suboptimal ultrasound image quality preventing reliable feature extraction (e.g., severe motion artifacts or incomplete scanning planes); (4) Inability to accurately match the ultrasound-targeted nodule with the pathological specimen (e.g., in cases of severe Hashimoto’s thyroiditis).

### Data collection

Collected clinicopathological parameters included gender, age, BRAF V600E mutation status, extrathyroidal extension, CLNM status, HT, and preoperative laboratory data including TRAB (Thyroid Stimulating Hormone Receptor Antibody), TPOAB (Thyroid Peroxidase Antibody), TGAB (Thyroglobulin Antibody), PTH (Parathyroid Hormone), CEA (Carcinoembryonic Antigen), TG (Thyroglobulin), FT3 (Free Triiodothyronine), FT4 (Free Thyroxine), and TSH (Thyroid Stimulating Hormone). Preoperative laboratory data were obtained from the patients’ electronic medical records. Notably, markers such as CEA were collected; these were not measured specifically for the evaluation of PTMC, but were part of our institution’s standardized comprehensive preoperative tumor screening panel to rule out medullary thyroid carcinoma or other concurrent malignancies prior to surgery. The diagnosis of HT was confirmed by final postoperative histopathology, supported by preoperative elevated serum thyroid autoantibodies and typical sonographic features.

**Figure 2:**
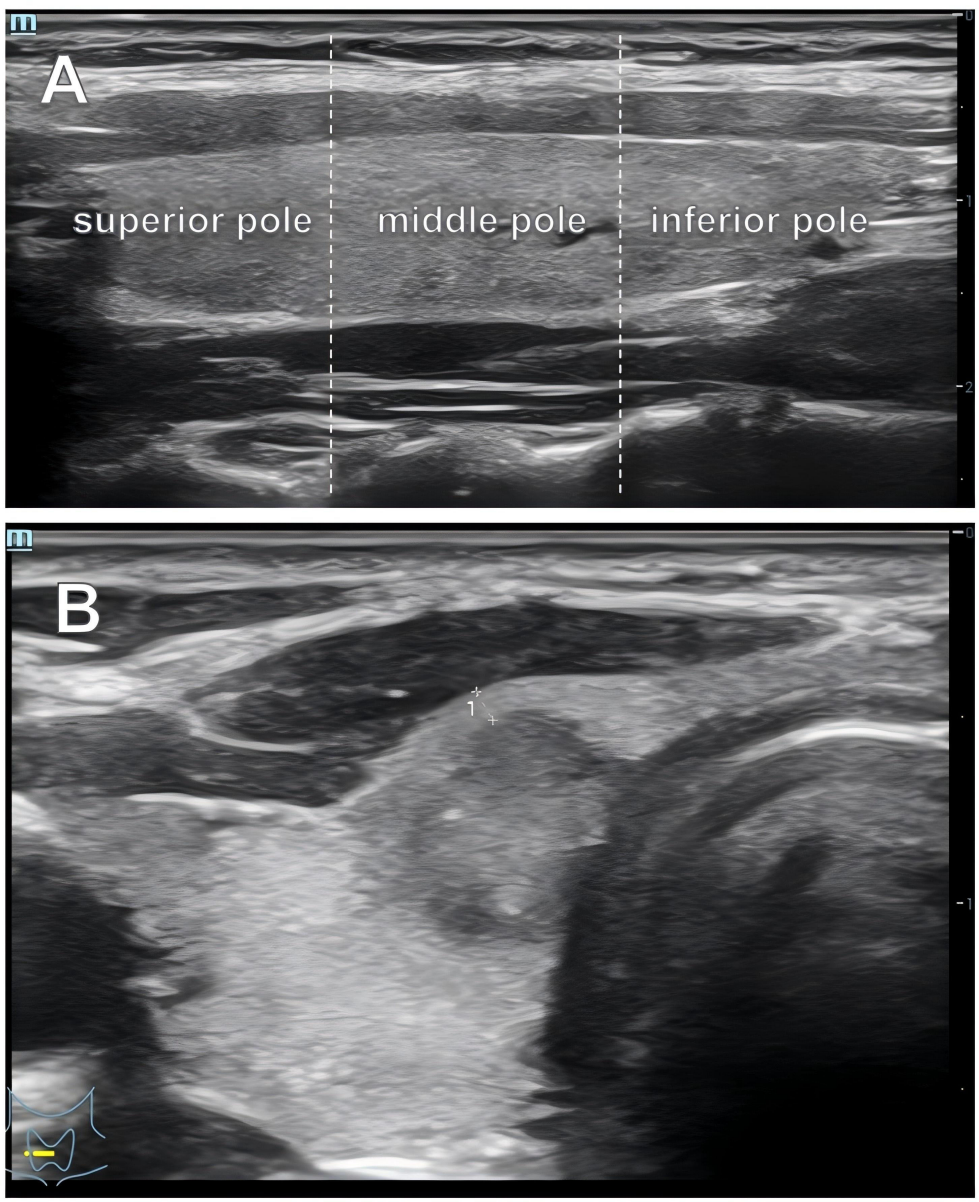
Lesion spatial localization method. **(A)** Division of the thyroid gland into the superior pole, middle pole, and inferior pole in the longitudinal view. **(B)** Schematic diagram illustrating a lesion adjacent to the thyroid capsule.

Preoperative ultrasonographic features included lesion volume, maximum diameter, echogenicity, margin, shape, aspect ratio, calcification type, vascularity, presence of suspicious central lymph nodes, and presence of suspicious lateral lymph nodes. Based on the spatial location of the lesion within the thyroid gland, the thyroid lobes were divided into the isthmus, superior pole, middle pole, and inferior pole. The relationship between the lesion and the thyroid capsule was recorded and categorized as adjacent to the anterior capsule, adjacent to the posterior capsule, or not adjacent to the capsule. Under dynamic ultrasound scanning of the thyroid gland, a lesion with a minimum distance of <2 mm from the thyroid capsule was defined as “adjacent” (**Figure 2**).

### Ultrasonographic feature analysis

All patients underwent thyroid and cervical lymph node ultrasound examination within one month before surgery. During the examination, patients were in the supine position with the neck fully exposed. Examinations were performed using Mindray, GE, or Philips ultrasound systems equipped with L12-5 high-frequency probes (frequency range 5–12 MHz). Six sonographers with over five years of experience in ultrasound imaging, blinded to the pathological results, independently assessed and recorded the ultrasonographic features. In case of disagreement, consultation was held with other sonographers to reach a final consensus.

### BRAF V600E mutation analysis

BRAF V600E mutation detection was performed using a commercially available kit from BIO-RAD (California, USA). Target DNA was extracted, and digital PCR was conducted according to the kit instructions. Results were determined using the reading standards provided by the mutation detection kit.

### Data analysis

All data were statistically analyzed using IBM SPSS Statistics for Windows, Version 26.0 (IBM Corp., Armonk, NY, USA) and R software version 4.4.3 (The R Foundation for Statistical Computing). Continuous variables were expressed as median and interquartile range (IQR), and compared using the Mann-Whitney U test. Categorical variables were expressed as numbers and percentages, and compared using the Chi-square test or Fisher’s exact test. A P-value <0.05 was considered statistically significant.

The Least Absolute Shrinkage and Selection Operator (LASSO) method was used in R software (version 4.4.3) to screen potential prognostic factors, minimizing classification error. A nomogram was plotted to visualize the predicted probability of CLNM for each patient. The discrimination performance of the model was evaluated using the receiver operating characteristic (ROC) curve. The calibration curve was validated using the bootstrap method (B = 1000 repetitions). Decision curve analysis (DCA) was used to calculate the net benefit at different threshold probabilities to evaluate the clinical utility of the model.

## Results

### Baseline characteristics

**Figure 3:**
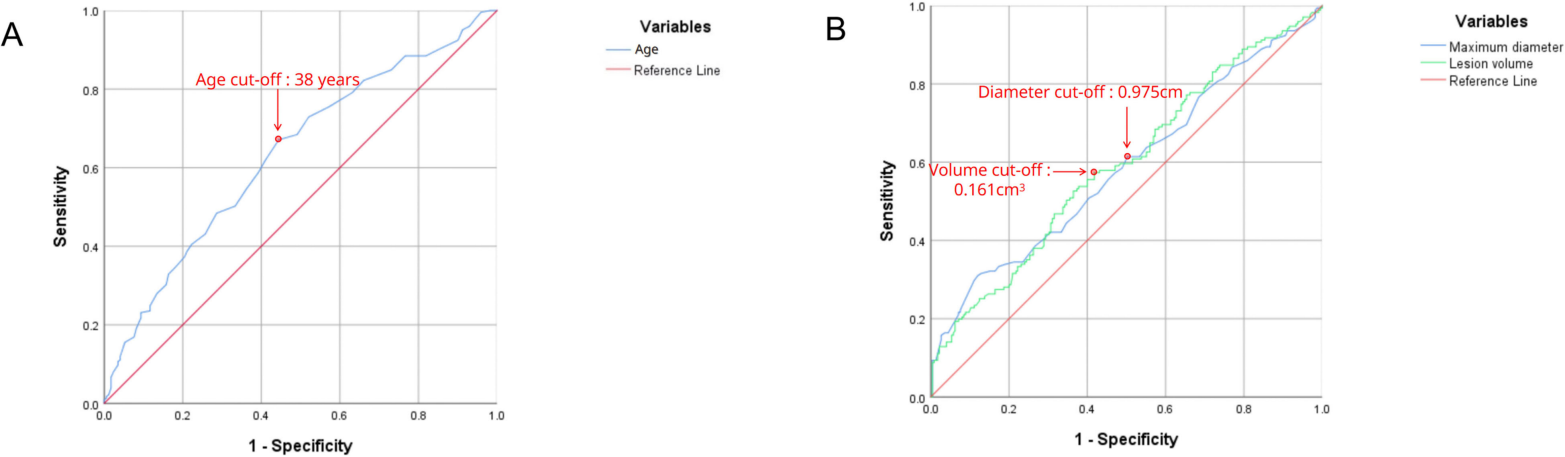
Receiver operating characteristic (ROC) curves for determining the optimal cut-off values of continuous baseline variables. **(A)** ROC curves for preoperative ultrasound maximal diameter and tumor volume. The statistically optimal cut-off value for the maximal diameter was calculated as 0.975 cm. However, for practical clinical application and taking into account the precision of ultrasound equipment, this value was approximated to 0.95 cm in subsequent categorical analyses. The optimal cut-off for tumor volume was identified as 0.161 cm³. **(B)** ROC curve for patient age, yielding an optimal cut-off of 38 years. All optimal thresholds (indicated by red circles) were determined based on the maximum Youden index.

A total of 432 patients (114 men and 318 women; mean age, 43.03 ± 12.17 years) were finally enrolled and randomly divided into a training set (n = 259) and a validation set (n = 173) in a 6:4 ratio for external validation. The baseline characteristics of the training and validation sets are shown in **Table 1**. Prior to further analyses, continuous baseline variables were evaluated using receiver operating characteristic (ROC) curves to determine their optimal categorical cut-off values based on the maximum Youden index. As illustrated in **Figure 3**, the mathematically optimal cut-offs were identified as 0.975 cm for preoperative ultrasound maximum diameter, 0.161 cm³ for tumor volume (**Figure 3A**), and 38 years for patient age (**Figure 3B**). For clinical applicability and ease of measurement, the statistical threshold of 0.975 cm for diameter was approximated to 0.95 cm in the subsequent feature categorization. meter Univariate analysis showed that age, HT, maximum diameter, margin, shape, aspect ratio, macrocalcifications, microcalcifications, vascularity, suspicious central/lateral lymph nodes, and location showed significant differences (all *p* < 0.05) between the CLNM-positive and CLNM-negative groups. No significant differences were found for gender, BRAF V600E mutation, extrathyroidal extension, and other listed laboratory markers.

**Table 1:** Characteristics of the unifocal PTMC training set and validation set.

Variables	Total	Training set	Validation set	*p* value
	(N = 432)	(N = 259)	(N = 173)	
Clinicopathological parameters				
Sex,n(%)				0.107
Male	114(26.39%)	68(26.25%)	46(26.59%)	
Female	318(73.61%)	191(73.75%)	127(73.41%)	
Age,n(%)				<0.001
≤38	187(43.2%)	119(45.94%)	68(39.3%)	
>38	245(56.7%)	140(54.05%)	105(60.6%)	
BRAF V600E,n(%)				0.824
Yes	412(95.3%)	247(95.3%)	165(95.3%)	
No	20(4.6%)	12(4.6%)	8(4.6%)	
Extrathyroidal extension,n(%)				0.682
Yes	281(65%)	169(65.2%)	112(64.7%)	
No	151(34.9%)	90(34.7%)	61(35.2%)	
HT,n(%)				0.004
Yes	142(32.87%)	86(33.2%)	56(32.37%)	
No	290(67.13%)	173(66.8%)	117(67.63%)	
Laboratory data				
TRAB (IU/L)				0.861
Median(IQR)	1.52 (1.02-2.15)	1.44 (0.97-1.89	1.63 (1.15-2.24)	
TPOAB (IU/ml)				0.996
Median(IQR)	6.83 (5.27-9.16)	7.12 (5.52-9.41)	6.77 (5.39-9.36)	
TGAB (IU/ml)				0.903
Median(IQR)	15.59 (10.37-18.92)	16.12 (11.02-19.53)	14.98 (10.48-18.39)	
PTH (pg/ml)				0.707
Median(IQR)	1.0 (1.0-1.0)	1.06 (1.0-1.1)	1.03 (1.0-1.1)	
CEA (ng/ml)				0.596
Median(IQR)	1.37 (0.67-2.47)	1.28 (0.59-2.20)	1.46 (0.72-2.58)	
TG (ng/ml)				0.513
Median(IQR)	19.13 (5.28-28.46)	18.8 (5.03-27.99)	19.54 (5.51-29.12)	
TSH (mIU/L)				0.213
Median(IQR)	2.45 (1.71-3.20)	2.51 (1.75-3.28)	2.39 (1.67-3.12)	
FT3 (pg/ml)				0.219
Median(IQR)	4.35 (3.95-4.72)	4.28 (3.88-4.65)	4.41 (4.02-4.78)	
FT4 (ng/dl)				0.443
Median(IQR)	13.29 (11.85-14.52)	13.41 (12.02-14.7)	12.97 (11.64-14.28)	
Ultrasonographic features				
Maximum diameter>0.95cm,n(%)				<0.001
Yes	86(19.9%)	50(19.3%)	36(20.8%)	
No	346(80.1%)	209(80.7%)	137(79.2%)	
Lesion volume>0.161cm^3^,n(%)				0.525
Yes	183(42.3%)	109(42.1%)	74(42.8%)	
No	249(57.6%)	150(57.9%)	99(57.2%)	
Echogenicity,n(%)				0.955
Isoechoic	2(0.4%)	1(0.4%)	1(0.6%)	
Hypoechoic	419(97%)	251(97%)	168(97.1%)	
Markedly echogenicity	8(1.8%)	5(1.9%)	3(1.7%)	
Mixed echogenicity	3(0.7%)	2(0.8%)	1(0.6%)	
Margin,n(%)				0.019
Well-defined	40(9.2%)	25(9.6%)	15(8.7%)	
ill-defined	119(27.5%)	71(27.4%)	48(27.7%)	
Poor-defined	273(63.2%)	163(62.9%)	110(40.3%)	
Shape,n(%)				0.03
Regular	38(8.8%)	23(8.9%)	15(8.7%)	
Mildly irregular	143(33.1%)	85(32.8%)	58(33.5%)	
Irregular	251(58.1%)	151(58.3%)	100(57.8%)	
Aspect ratio>1,n(%)				0.043
Yes	326(75.5%)	194(74.9%)	132(76.3%)	
No	106(24.5%)	65(25.1%)	41(23.7%)	
Macrocalcification,n(%)				0.001
Yes	61(14.1%)	33(12.7%)	28(16.2%)	
No	371(85.9%)	226(87.2%)	145(83.8%)	
Microcalcification,n(%)				<0.001
Yes	228(52.8%)	140(54.1%)	88(50.8%)	
No	204(47.2%)	119(45.9%)	85(49.1%)	
Vascularity,n(%)				0.05
Visible	189(43.8%)	111(42.8%)	78(45.1%)	
Unvisible	243(56.2%)	148(57.1%)	95(54.9%)	
Suspicious central lymph nodes,n(%)				<0.001
Visible	50(11.6%)	30(11.6%)	20(11.5%)	
Unvisible	382(88.4%)	229(88.4%)	153(88.4%)	
Suspicious lateral lymph nodes,n(%)				<0.001
Visible	37(8.5%)	22(8.5%)	15(8.6%)	
Unvisible	395(91.4%)	237(91.5%)	158(91.3%)	
Location,n(%)				0.011
1	33(7.6%)	21(8.1%)	12(6.9%)	
2	48(11.1%)	31(11.9%)	17(9.8%)	
3	53(12.2%)	34(13.1%)	19(10.9%)	
4	49(11.3%)	31(11.9%)	18(10.4%)	
5	138(31.9%)	78(30.1%)	60(34.7%)	
6	72(16.7%)	41(15.8%)	31(17.9%)	
7	12(2.8%)	6(2.3%)	6(3.4%)	
8	27(6.2%)	17(6.5%)	10(5.8%)	

Locations 1-8 are defined as: 1: Superior/middle poles not adjacent to capsule; 2: Superior pole adjacent to anterior capsule; 3: Superior pole adjacent to posterior capsule; 4: Middle pole adjacent to anterior capsule; 5: Middle/inferior poles adjacent to posterior capsule; 6: Inferior pole adjacent to anterior capsule; 7: Inferior pole not adjacent to capsule; 8: Isthmus.

HT, Hashimoto’s thyroiditis; IQR, interquartile range; TRAB, Thyroid Stimulating Hormone Receptor Antibody; TPOAB, Thyroid Peroxidase Antibody; TGAB, Thyroglobulin Antibody; PTH, Parathyroid Hormone; CEA, Carcinoembryonic Antigen; TG, Thyroglobulin; TSH, Thyroid Stimulating Hormone; FT3, Free Triiodothyronine; FT4, Free Thyroxine.

To further elucidate the impact of spatial location, we calculated the CLNM rate for each location (**Table 2**). Based on these rates, high-risk locations were defined as Location 8 (Isthmus; CLNM rate 63%), Location 5 (Middle/inferior poles adjacent to posterior capsule; 46%), Location 4 (Middle pole adjacent to anterior capsule; 47%), and Location 3 (Superior pole adjacent to posterior capsule; 43%). Conversely, low-risk locations were Location 1 (Superior/middle poles, not adjacent to capsule; 24%) and Location 2 (Superior pole adjacent to anterior capsule; 23%). This clear gradient in risk (ranking: Location 8 > Location 4 > Location 5 > Location 3 > Other locations) provided the foundation for incorporating spatial location as a key variable in the subsequent prediction model.

**Table 2:** Central lymph node metastasis rate for each location.

Location	Description	CLNM(+),N	Total,N	CLNM rate,%
1	Superior/middle poles not adjacent to capsule	8	33	24%
2	Superior pole adjacent to anterior capsule	11	48	23%
3	Superior pole adjacent to posterior capsule	23	53	43%
4	Middle pole adjacent to anterior capsule	23	49	47%
5	Middle/inferior poles adjacent to posterior capsule	64	138	46%
6	Inferior pole adjacent to anterior capsule	30	72	42%
7	Inferior pole not adjacent to capsule	5	12	42%
8	Isthmus	17	27	63%

CLNM, Central Lymph Node Metastasis. Feature selection.

The multivariate logistic regression model (incorporating covariates such as age, maximum lesion diameter and microcalcifications) further indicated that the location was an independent predictor of CLNM (*p* < 0.05). Concurrently, it was concluded that age ≤38 years, maximum tumor diameter >0.95 cm, microcalcifications, ultrasonographically suspicious central lymph nodes, and ultrasonographically suspicious lateral lymph nodes were independent risk factors for CLNM in PTMC patients. HT was a protective factor. The distribution characteristics of each factor in the CLNM (+) and CLNM (-) groups are listed in **Table 3**.

**Table 3:** Baseline characteristics of patients with and without central lymph node metastasis (clnm) in the training and validation sets.

Variables	Training set (N=259)		Validation set (N=173)		Total (N=432)		*p* value
	CLNM (+)	CLNM (-)	CLNM (+)	CLNM (-)	CLNM (+)	CLNM (-)	
Age,n (%)							<0.001
≤38	60 (58.25%)	59 (37.82%)	39 (50%)	29 (30.53%)	99 (54.7%)	88 (35.1%)	
>38	43 (41.75%)	97 (62.18%)	39 (50%)	66 (69.47%)	82 (45.3)	163 (64.94%)	
HT,n (%)							0.004
Yes	31 (30.1%)	55 (35.26%)	20 (25.64%)	36 (37.89%)	51 (28.18%)	91 (36.25%)	
No	72 (69.9%)	101 (64.74%)	58 (74.36%)	59 (62.11%)	130 (71.82%)	160 (63.75%)	
Maximum diameter>0.95cm,n (%)							<0.001
Yes	30 (29.13%)	20 (12.82%)	24 (30.77%)	12 (12.63%)	54 (29.83%)	32 (12.75%)	
No	73 (70.87%)	136 (87.18%)	54 (69.23%)	83 (87.37%)	127 (70.17%)	219 (87.25%)	
Margin,n (%)							0.379
Well-defined	4 (3.88%)	20 (12.82%)	4 (5.13%)	12 (12.63%)	8 (4.42%)	32 (12.75%)	
ill-defined	27 (26.21%)	45 (28.85%)	20 (25.64%)	28 (29.47%)	47 (25.97%)	73 (29.08%)	
Poor-defined	72 (69.9%)	91 (58.33%)	54 (69.23%)	55 (57.89%)	126 (69.61%)	146 (58.17%)	
Shape,n (%)							0.418
Regular	4 (3.88%)	20 (12.82%)	3 (3.85%)	11 (11.58%)	7 (3.87%)	31 (12.35%)	
Mildly irregular	33 (32.04%)	53 (33.97%)	26 (33.33%)	31 (32.63%)	59 (32.6%)	84 (33.47%)	
Irregular	66 (64.08%)	83 (53.21%)	49 (62.82%)	53 (55.79%)	115 (63.54%)	136 (54.18%)	
Aspect ratio>1,n (%)							0.187
Yes	83 (80.58%)	112 (71.79%)	64 (82.05%)	67 (70.53%)	147 (81.22%)	179 (71.31%)	
No	20 (19.42%)	44 (28.21%)	14 (17.95%)	28 (29.47%)	34 (18.78%)	72 (28.69%)	
Macrocalcification,n (%)							0.038
Yes	14 (13.59%)	19 (12.18%)	13 (16.67%)	15 (15.79%)	27 (14.92%)	34 (13.55%)	
No	89 (86.41%)	137 (87.82%)	65 (83.33%)	80 (84.21%)	154 (85.08%)	217 (86.45%)	
Microcalcification,n (%)							0.003
Yes	66 (64.08%)	74 (47.44%)	49 (62.82%)	39 (41.05%)	115 (63.54%)	113 (45.02%)	
No	37 (35.92%)	82 (52.56%)	29 (37.18%)	56 (58.95%)	66 (36.46%)	138 (54.98%)	
Vascularity,n (%)							0.657
Visible	48 (46.6%)	63 (40.38%)	41 (52.56%)	37 (38.95%)	89 (49.17%)	100 (39.84%)	
Unvisible	55 (53.4%)	93 (59.62%)	37 (47.44%)	58 (61.05%)	92 (50.83%)	151 (60.16%)	
Suspicious central lymph nodes,n (%)							<0.001
Visible	20 (19.42%)	10 (6.41%)	15 (19.23%)	5 (5.26%)	35 (19.34%)	15 (5.98%)	
Unvisible	83 (80.58%)	146 (93.59%)	63 (80.77%)	90 (94.74%)	146 (80.66%)	236 (94.02%)	
Suspicious lateral lymph nodes,n (%)							0.005
Visible	15 (14.56%)	7 (4.49%)	12 (15.38%)	3 (3.16%)	27 (14.92%)	10 (3.98%)	
Unvisible	88 (85.44%)	149 (95.51%)	66 (84.62%)	92 (96.84%)	154 (85.08%)	241 (96.02%)	
Location,n (%)							0.048
1	4 (3.88%)	17 (10.9%)	4 (5.13%)	8 (8.42%)	8 (4.42%)	25 (9.96%)	
2	7 (6.8%)	24 (15.38%)	4 (5.13%)	13 (13.68%)	11 (6.08%)	37 (14.74%)	
3	16 (15.53%)	18 (11.54%)	7 (8.97%)	12 (12.63%)	23 (12.71%)	30 (11.95%)	
4	14 (13.59%)	17 (10.9%)	9 (11.54%)	9 (9.47%)	23 (12.71%)	26 (10.36%)	
5	33 (32.04%)	45 (28.85%)	31 (39.74%)	29 (30.53%)	64 (35.36%)	74 (29.48%)	
6	14 (13.59%)	27 (17.31%)	16 (20.51%)	15 (15.79%)	30 (16.57%)	42 (16.73%)	
7	4 (3.88%)	2 (1.28%)	1 (1.28%)	5 (5.26%)	5 (2.76%)	7 (2.79%)	
8	11 (10.68%)	6 (3.85%)	6 (7.69%)	4 (4.21%)	17 (9.39%)	10 (3.98%)	

Factors with *p* < 0.05 in the univariate analysis were adopted for the multivariable analysis;.

Locations 1-8 are defined as: 1: Superior/middle poles not adjacent to capsule; 2: Superior pole adjacent to anterior capsule; 3: Superior pole adjacent to posterior capsule; 4: Middle pole adjacent to anterior capsule; 5: Middle/inferior poles adjacent to posterior capsule; 6: Inferior pole adjacent to anterior capsule; 7: Inferior pole not adjacent to capsule; 8: Isthmus.

### Development of the nomogram.

**Figure 4:**
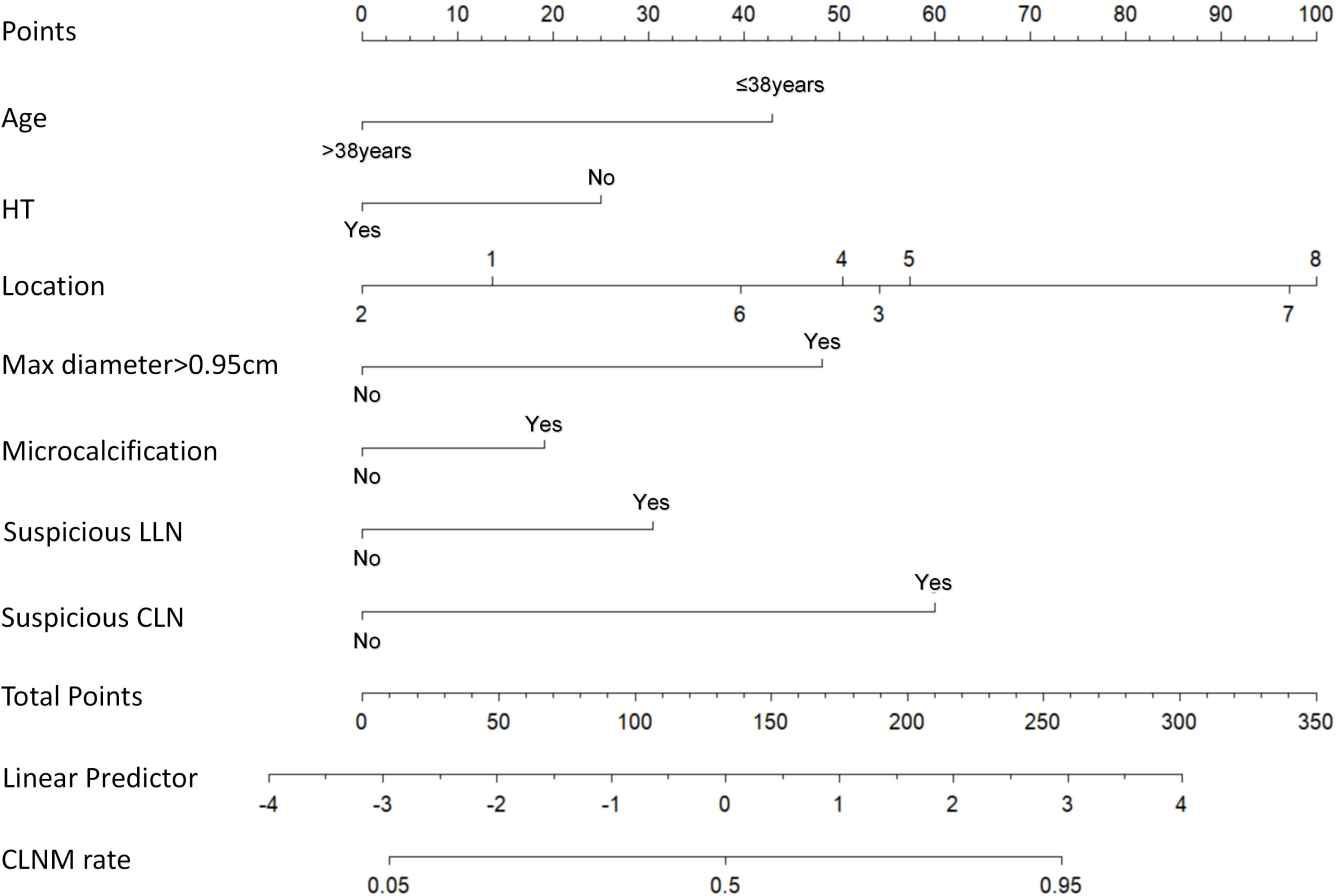
The nomogram for predicting CLNM in unifocal PTMC patients. To use this nomogram, draw a vertical line from the patient’s value on each of the 7 predictor axes up to the “Points” scale to obtain the individual score. Sum all 7 scores to get the “Total Points,” then draw a vertical line down to the bottom axis to determine the estimated CLNM probability. The “Linear Predictor” axis represents the mathematical model value and can be bypassed in routine clinical use. tumor locations (1–8): 1: Superior/middle poles not adjacent to capsule; 2: Superior pole adjacent to anterior capsule; 3: Superior pole adjacent to posterior capsule; 4: Middle pole adjacent to anterior capsule; 5: Middle/inferior poles adjacent to posterior capsule; 6: Inferior pole adjacent to anterior capsule; 7: Inferior pole not adjacent to capsule; 8: Isthmus. (Abbreviations: PTMC, papillary thyroid microcarcinoma; HT, Hashimoto’s thyroiditis; LLN, lateral lymph nodes; CLN, central lymph nodes.).

**Figure 5:**
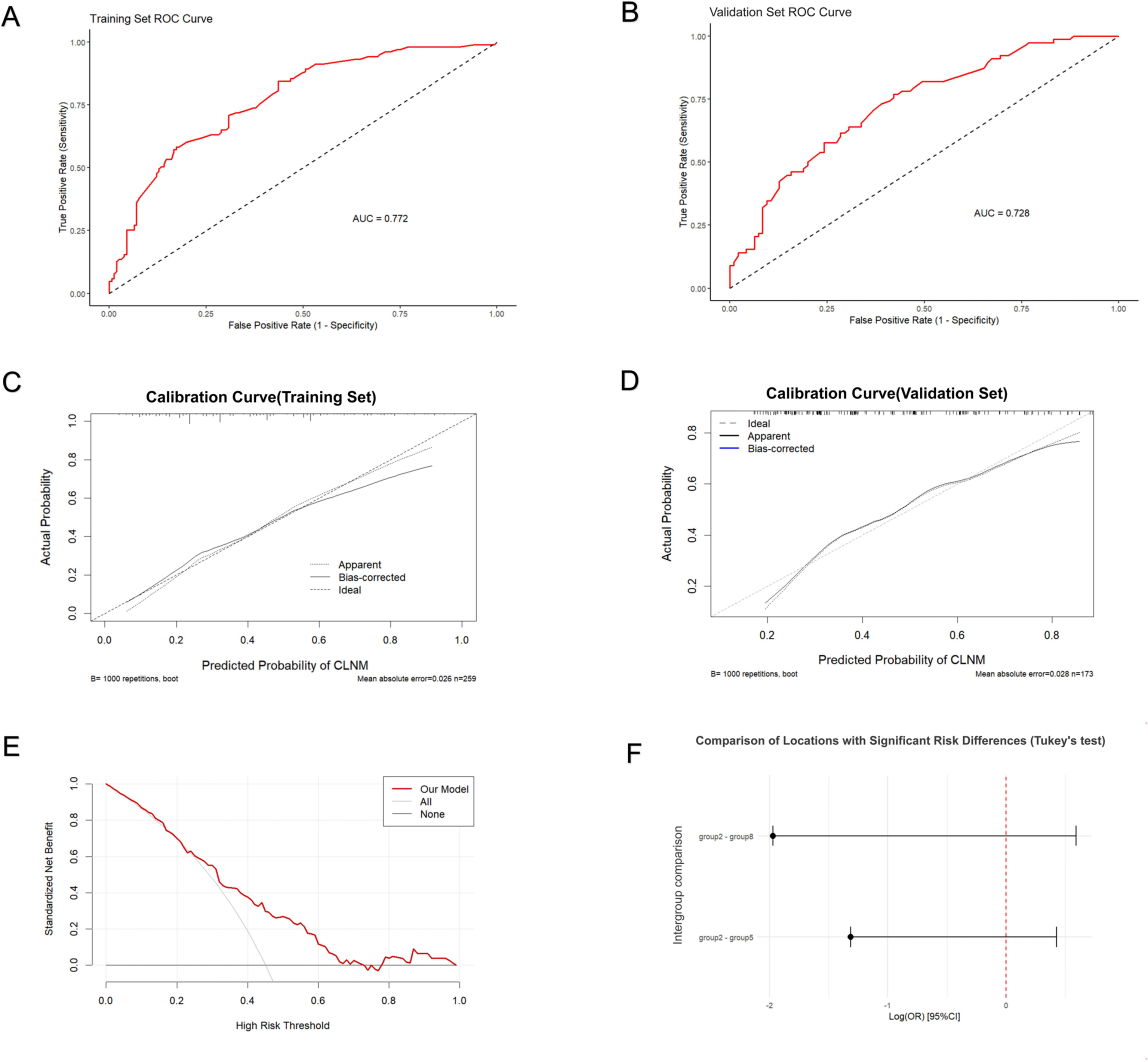
Development and performance assessment of the CLNM prediction nomogram. **(A, B)** Receiver operating characteristic (ROC) curves of the nomogram in the **(A)** training set (AUC = 0.772, 95% CI: 0.715-0.830) and **(B)** validation set (AUC = 0.728, 95% CI: 0.653-0.803). **(C, D)** Calibration curves of the nomogram for the **(C)** training set (Bootstrap repetitions=1000; mean absolute error=0.026; Hosmer-Lemeshow test *p* = 0.337) and **(D)** validation set (Bootstrap repetitions=1000; mean absolute error=0.028). The dashed line represents the ideal fit, and the solid line represents the apparent or bias-corrected performance. **(E)** Decision curve analysis (DCA) for the nomogram. The net benefit of using the nomogram for clinical decision-making is plotted against the threshold probability. **(F)** Statistical validation of risk location definitions via Tukey’s *post-hoc* test following one-way ANOVA. The comparisons demonstrate significant differences in CLNM risk between the defined high-risk and low-risk zones (*p* < 0.05).

The 7 independent predictors incorporated into the final nomogram were strictly selected via a standardized three-step feature selection workflow to prevent overfitting: univariate logistic regression to screen candidate variables (P < 0.05), LASSO regression to eliminate collinearity and reduce dimensionality, and multivariate logistic regression to confirm the final independent predictors with statistical significance (P < 0.05). The constructed nomogram is presented in **Figure 4**. After adjusting for confounding factors, the ‘risk score’ for each location in the nomogram was positively correlated with the probability of CLNM. Specifically: Location 8 (Isthmus) and Location 5 (Middle/inferior poles adjacent to posterior capsule) corresponded to the highest scores in the nomogram, indicating the highest independent risk; The score for Location 2 (Superior pole adjacent to anterior capsule) was significantly lower than that for Location 1 (Superior/middle poles not adjacent to capsule) (*p* < 0.05), indicating a lower net risk for Location 2 after controlling for other factors; The score for Location 7 (Inferior pole not adjacent to capsule) was significantly higher than that for Location 1 and Location 2 (*p* < 0.05), suggesting that the inferior pole location itself carries a higher independent risk even without capsular adjacency. ROC curves were plotted to predict the nomogram’s effect (**Figures 5A, B**). The area under the ROC curve (AUC) was 0.772 (95% CI: 0.715–0.830) for the training set and 0.728 (95% CI: 0.653–0.803) for the validation set, indicating good predictive discrimination of the model in both sets. According to the established statistical criteria by Hosmer and Lemeshow (23), an AUC between 0.7 and 0.8 represents an acceptable level of discrimination. Accordingly, our model’s AUCs of 0.772 and 0.728 demonstrate a reasonable discriminative ability, which we believe can serve as a helpful quantitative reference in clinical practice. The calibration curves showed high consistency between the predicted and actual probabilities (**Figures 5C, D**). The Hosmer-Lemeshow goodness-of-fit test yielded a P-value of 0.337, indicating a good fit of the model. To better illustrate the clinical utility of this nomogram, we present a clinical case: a 35-year-old patient (Age ≤ 38 years, approx. 43 points), without Hashimoto’s thyroiditis (HT: No, approx. 25 points), presenting with a tumor located at the middle pole adjacent to the anterior capsule (Location 4, approx. 50 points). Preoperative ultrasound revealed a maximum diameter of 0.98 cm (Max diameter >0.95cm, approx. 48 points), the presence of microcalcifications (Microcalcification: Yes, approx. 19 points), but no suspicious lymph nodes in either the central or lateral compartments (Suspicious CLN/LLN: No, 0 points). Summing these individual scores yielded a Total Points score of 185 (43 + 25 + 50 + 48 + 19 + 0+0). By drawing a vertical line downward from 185 on the ‘Total Points’ axis to the ‘CLNM rate’ axis, the nomogram estimated an approximately 80% probability of central lymph node metastasis for this cN0 patient. Notably, the postoperative pathology of this patient confirmed the presence of CLNM. This representative case effectively demonstrates how the model can identify high-risk individuals among clinical cN0 patients, thereby guiding individualized surgical decision-making, such as considering prophylactic central lymph node dissection.

### Clinical utility and zonal validation

Decision curve analysis (**Figure 5E**) showed that using this nomogram to predict CLNM risk provided net clinical benefit within a threshold probability range of 0.2–0.8. As shown by the Tukey *post-hoc* pairwise comparisons (**Figure 5F**), significant differences in CLNM risk were observed between Location 2, the lowest-risk zone, and Location 8, the highest-risk zone, as well as Location 5. This finding provides direct support for defining Location 2 as a protective location and Locations 5 and 8 as high-risk locations.

## Discussion

Accurately identifying PTMC patients at high risk for CLNM is key to formulating individualized surgical strategies. This study innovatively integrated tumor spatial location with clinical and ultrasonographic features to successfully construct and validate a nomogram model with good predictive performance (training set AUC 0.772), providing a more comprehensive visual tool for preoperative accurate assessment of CLNM risk.

Multivariate analysis confirmed that age ≤38 years, maximum tumor diameter >0.95 cm, microcalcifications, and ultrasonographically suspicious central or lateral lymph nodes were independent risk factors for CLNM, consistent with most previous studies (22, 24, 25). Microcalcifications, a classic malignant ultrasonographic feature, showed a confirmed association with CLNM in our model (26). Currently, microcalcifications are considered the sonographic correlate of psammoma body formation. The latter may promote lymphatic metastasis through mechanisms such as calcification deposition, metabolic reprogramming, and synergy with BRAF mutations (27–29), although the precise mechanisms require further investigation. Additionally, ultrasonographic suspicion of lymph node abnormality is the most direct imaging clue for CLNM. Although breaching the thyroid capsule is not strictly essential for lymphatic metastasis—given that tumor cells can spread directly via the rich intra-thyroidal lymphatic network—tumors that breach or lie adjacent to the capsule gain earlier access to the abundant extracapsular lymphatic plexus, which significantly elevates the risk of metastasis to the central and subsequently lateral neck compartments (30, 31). Notably, coexisting HT exhibited a protective effect, potentially related to the local immune microenvironment it creates, inhibiting tumor metastasis (32). However, this finding should be interpreted with caution. As highlighted by epidemiological perspectives (33, 34), this reduced risk may be partially attributed to surveillance bias (or lead-time bias). Patients with HT typically undergo more frequent medical surveillance and routine thyroid ultrasounds due to chronic thyroiditis or hypothyroidism. Consequently, their PTMCs are more likely to be detected at an earlier, more indolent stage before lymphatic metastasis occurs. Therefore, the protective role of HT in our nomogram reflects a real-world clinical scenario of earlier detection rather than an absolute biological inhibition of tumor spread. Furthermore, this study found no significant association between BRAF V600E mutation and CLNM, consistent with the report by XUE et al. (35), further suggesting that BRAF mutation may have limited value in predicting lymph node metastasis in PTMC.

The incorporation of tumor spatial location is a central highlight of this study. We found that tumors located in the isthmus or in the middle/inferior pole adjacent to the posterior capsule carried a significantly higher risk of CLNM. This finding not only confirms spatial location as an independent predictor of CLNM but also elucidates its underlying dynamistic mechanisms from an anatomico-physiological perspective. The isthmus, functioning as a lymphatic hub with a dense network and bidirectional drainage characteristics, facilitates tumor cell dissemination to bilateral central compartments. Once tumor cells breach the basement membrane, they can drain with nearly equal probability into the prelaryngeal and pretracheal lymph nodes on either side. Lymphatic drainage from the middle and inferior poles primarily converges towards the central compartment. When a lesion is intimately close to the posterior capsule (e.g., <2 mm), it signifies a proximate spatial relationship with the rich extraglandular lymphatic plexus (36, 37), where lymph fluid collects into the paratracheal and para-recurrent laryngeal nodal networks. This adjacency significantly reduces the physical barrier for tumor cells to complete the metastatic cascade of invasion-migration-colonization (38), markedly increasing the risk of early capsular invasion and lymphatic spread. Conversely, lesions situated in the superior/middle pole and close to the anterior capsule exhibited a protective effect, likely attributable to their relative isolation from the main central lymphatic drainage pathways. Lymphatics from this area must traverse a longer course to reach the primary trunks and are anatomically buffered by structures like the thyroid cartilage, forming a natural barrier that objectively impedes tumor dissemination (39). This explains the impact of spatial location on metastasis from an anatomical standpoint and provides a more precise target for ultrasound assessment.

This study has several limitations. First, the retrospective, single-center design of this study ensured internal data consistency but may have introduced selection bias, thereby limiting the generalizability of our findings. Therefore, future prospective, multi-center studies are necessary to further validate the broader applicability of our model. Second, the current explanations regarding the role of tumor location and Hashimoto’s thyroiditis remain inferential. We plan to utilize preoperative contrast-enhanced ultrasound and postoperative immunohistopathological analysis to directly verify whether tumors in high-risk locations possess a more aggressive lymph-invasive biological phenotype. Furthermore, as discussed earlier, the observed protective effect of Hashimoto’s thyroiditis in our retrospective cohort is heavily confounded by clinical detection bias due to frequent screening. Concurrently, we aim to disentangle this screening effect from true biological tumor inhibition by analyzing the peritumoral lymphatic vessel status and local immune microenvironment in future prospective studies. Third, heterogeneity in ultrasound equipment across centers and the inherent subjectivity of image interpretation are unavoidable challenges. Although we sought to minimize bias by establishing inter-observer consensus, the future integration of computer-aided diagnosis systems for objective quantification represents a crucial direction for model optimization. Fourth, regarding the molecular signature of our samples, although we utilized highly sensitive digital PCR for BRAF V600E detection, the retrospective nature of our data means we must consider the potential for DNA degradation in Formalin-Fixed Paraffin-Embedded (FFPE) tissues over time. This degradation could theoretically lead to inadequate molecular profiling or false-negative results in a subset of older samples, which might partially account for the lack of statistical significance regarding the BRAF mutation in our cohort. Finally, a methodological note regarding tumor size measurement should be acknowledged. In our study, the inclusion criterion for PTMC was strictly defined by the postoperative pathological maximum diameter (≤1.0 cm), which is the clinical gold standard. However, the predictive analyses and ROC curves were based on preoperative ultrasound measurements. It has been reported in previous literature (40) that ultrasound measurements may sometimes overestimate actual tumor size compared to final pathology, potentially due to the sonographic halo effect or tissue shrinkage following formalin fixation. As our study strictly adhered to the pathological diameter (≤1.0 cm) for inclusion, a small proportion of patients with preoperative ultrasound measurements marginally exceeding 1.0 cm were ultimately included upon pathological confirmation of PTMC. We acknowledge this inherent discrepancy between *in vivo* imaging and ex vivo pathology, which might account for why our calculated optimal cut-off for ultrasound diameter (0.975 cm) approaches the 1.0 cm threshold.

## Conclusion

This study successfully developed and validated a nomogram model integrating clinical, ultrasonographic, and innovative spatial location features. The model demonstrated good predictive performance, particularly clarifying that specific tumor spatial locations (e.g., isthmus, middle/inferior pole adjacent to the posterior capsule) are important independent predictors of CLNM. This tool aids in the preoperative identification of high-risk PTMC patients, provides an objective basis for formulating individualized surgical plans, and is expected to help reduce overtreatment and optimize the utilization of medical resources.

## Data Availability

The original contributions presented in the study are included in the article/supplementary material. Further inquiries can be directed to the corresponding author.
